# B Cell Lymphoma 2: A Potential Therapeutic Target for Cancer Therapy

**DOI:** 10.3390/ijms221910442

**Published:** 2021-09-28

**Authors:** Manzar Alam, Sabeeha Ali, Taj Mohammad, Gulam Mustafa Hasan, Dharmendra Kumar Yadav, Md. Imtaiyaz Hassan

**Affiliations:** 1 Centre for Interdisciplinary Research in Basic Sciences, Jamia Millia Islamia, Jamia Nagar, New Delhi 110025, India; manzar987@gmail.com (M.A.); ra.sabeeha@jmi.ac.in (S.A.); taj144796@st.jmi.ac.in (T.M.); 2 Department of Biochemistry, College of Medicine, Prince Sattam Bin Abdulaziz University, Al-Kharj 11942, Saudi Arabia; mgulam@gmail.com; 3 Department of Pharmacy and Gachon Institute of Pharmaceutical Science, College of Pharmacy, Gachon University, Hambakmoeiro 191, Yeonsu-gu, Incheon 21924, Korea

**Keywords:** B cell lymphoma 2, cancers, apoptosis, inhibitors, clinical trials, targeted therapy

## Abstract

Defects in the apoptosis mechanism stimulate cancer cell growth and survival. B cell lymphoma 2 (Bcl-2) is an anti-apoptotic molecule that plays a central role in apoptosis. Bcl-2 is the founding constituent of the Bcl-2 protein family of apoptosis controllers, the primary apoptosis regulators linked with cancer. Bcl-2 has been identified as being over-expressed in several cancers. Bcl-2 is induced by protein kinases and several signaling molecules which stimulate cancer development. Identifying the important function played by Bcl-2 in cancer progression and development, and treatment made it a target related to therapy for multiple cancers. Among the various strategies that have been proposed to block Bcl-2, BH3-mimetics have appeared as a novel group of compounds thanks to their favorable effects on many cancers within several clinical settings. Because of the fundamental function of Bcl-2 in the regulation of apoptosis, the Bcl-2 protein is a potent target for the development of novel anti-tumor treatments. Bcl-2 inhibitors have been used against several cancers and provide a pre-clinical platform for testing novel therapeutic drugs. Clinical trials of multiple investigational agents targeting Bcl-2 are ongoing. This review discusses the role of Bcl-2 in cancer development; it could be exploited as a potential target for developing novel therapeutic strategies to combat various types of cancers. We further highlight the therapeutic activity of Bcl-2 inhibitors and their implications for the therapeutic management of cancer.

## 1. Introduction

Bcl-2 is an anti-apoptotic protein that is associated with several cancer progression [1,2]. Bcl-2 was the first protein to be documented among the Bcl-2 family proteins [3]. It was the first gene exhibited to promote prolonged cell survival and growth rather than enhanced proliferation, which revealed that inhibition of cell death is vital in tumorigenesis [4]. Bcl-2 represses apoptosis by inhibiting cytochrome c (cyt-*c*) release from the mitochondria, preventing activation of caspases responsible for apoptosis [5]. Elevated expression of Bcl-2 is involved in many cancer progressions [6]. The increased expression of Bcl-2 is linked with advanced stage neoplasms and poor differentiation [7]. Bcl-2 plays a vital role in angiogenesis and cancer growth [8]. Apoptosis is the most important mechanism of cell death in response to tumor therapies [9]. Targeting the Bcl-2 protein may improve apoptosis by chemotherapeutic agents [10,11]. Bcl-2 inhibitors have exhibited promising effects in several malignancies with a single drug and combination with other drugs since Bcl-2 is a potential therapeutic target for malignancies [12].

Bcl-2 is the most significant protein, its overexpression and phosphorylation may be linked to regulation of cell growth, cell cycle, proliferation, DNA repair, and tumorigenesis. Elevated expression of Bcl-2 protein has been found in several human cancers [13,14]. Bcl-2 participates in an oncogenic function via survival signaling pathways and its role on the mitochondrial membrane. Several signaling molecules regulate Bcl-2. The phosphorylation of Bcl-2 on Ser70 through growth factor-activated protein kinases triggers the anti-apoptotic function of Bcl-2 [15,16]. p53 regulates Bcl-2 family proteins and hence controls mitochondria-mediated apoptosis [17]. p53 is a controller of the expression of the *Bcl-2* gene. It increases Bcl-2 protein expression [18,19,20]. Overexpression of Bcl-2 showed more activation of signaling molecules in pancreatic cancer cells [21]. Signaling pathways play a major role in controlling the Bcl-2 family of proteins that initiates cell growth/survival via activation of Bcl-2 [22,23]. 

Here, this review article highlights the present findings on the role of Bcl-2 in the progression of various cancers and its significance as a therapeutic target. We also discussed a comprehensive study of Bcl-2 inhibitors as a therapeutic target for cancer therapy. This investigation summarizes the potential of Bcl-2-inhibitors in the treatment of cancer and novel plans to utilize these inhibitors in pre-clinical and clinical trial applications.

## 2. Bcl-2 Family Proteins Mediated Apoptosis

**Figure 1:**
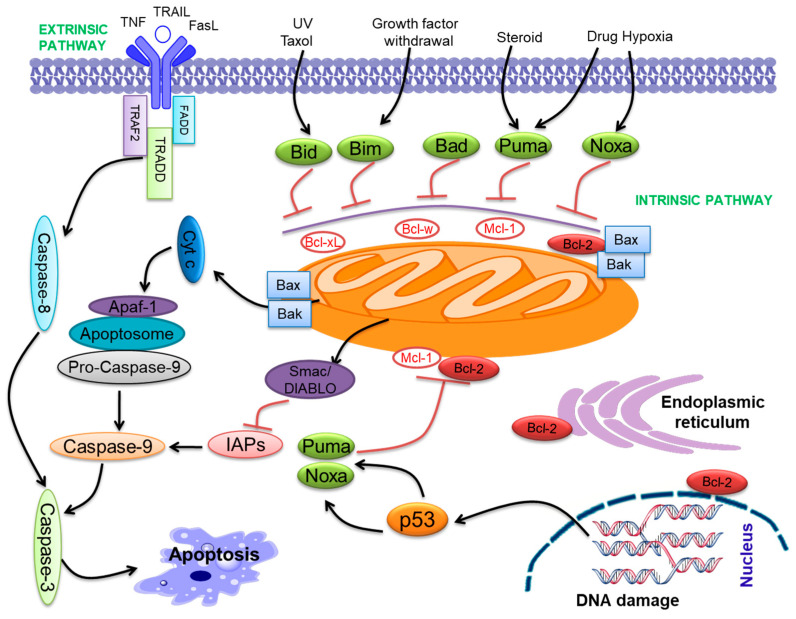
The intrinsic and extrinsic pathways of apoptosis. The two pathways that initiate apoptosis are the extrinsic (or death receptor initiated by ligand binding and subsequent activation of caspase-8) pathway and intrinsic pathway (driven by Bcl-2 family proteins, release cyt-*c* and activation of caspase-9) of apoptosis. Both pathways lead to a common apoptosis pathway by activating caspases-3, -6, and -7, triggering apoptosis. Bcl-2 family proteins are primarily localized to mitochondria and present on the ER and the perinuclear membrane in hematopoietic cells.

Apoptosis is the complex and fine-regulated appearance of programmed cell death. It takes part in vital functions such as embryogenesis, immunity, tissue development, and maintenance of homeostasis. Hence, excessive or insufficient cell death may lead to many pathological conditions like cancer [24,25]. Apoptosis is an important program of a living cell [24,26]. Two pathways initiate apoptosis, the extrinsic pathway and the intrinsic pathway (Figure 1). The extrinsic pathway starts with the activation of particular death receptors and ligand receptors. These ligands contain Apo2L/TRAIL and CD95L/FasL, which bind with DR4/DR5 (TRAIL-R1/R2) and CD95/Fas [27]. The intrinsic pathway of apoptosis is primarily triggered via the Bcl-2 family proteins [28]. Bcl-2 family proteins participate in p53-mediated apoptosis, which interacts with the Bcl-2 family proteins, reduces the mitochondrial membrane permeabilization (MMP), and releases cyt-c that activates caspase-9 [29,30,31,32]. The cyt-*c* and Apaf-1 binding activate pro-caspase-9, forming an “apoptosome” [33]. Both pathways activate caspase-3 and follow the apoptosis pathway (Figure 1) [34]. 

Bcl-2 family proteins include both pro-apoptotic (Bax, Bak, Bid, Bim, Bad, Bik, Puma, Noxa, etc.) and anti-apoptotic (Bcl-2, Bcl-xL, Bcl-w, Mcl-1, etc.) proteins [35,36]. Bcl-2 family proteins contribute to a common genetic region, the Bcl-2 homology (BH) domain, containing four conserved BH domains [32,37]. The Bcl-2 family proteins are categorized into anti-apoptotic proteins, pro-apoptotic multi-domain Bcl-2 proteins, and BH3-only proteins. The anti-apoptotic compose BH1-4 [38], while Bax and Bak compose BH1-3 and so-called ‘multi-domain’ pro-apoptotic proteins [38]. Bcl-2 family proteins were demonstrated to contribute to cancer progression [39,40]. All anti-apoptotic Bcl-2 proteins seem to play the role of oncoproteins, and pro-apoptotic proteins may have a role as tumor suppressors. These molecules perform important functions in cancer progression [41,42]. Further, the BH3 domain combines with the anti-apoptotic proteins, neutralizing them and initiating apoptosis [43]. The down-expression of pro-apoptotic proteins is linked with cancer progression [44]. Pro-apoptotic Bax was associated with oral cancer progression and drug resistance [45]. Anti-apoptotic Bcl-2 proteins play a major role in the progression of various neoplasms [46] and fuel rapid cell cycle regulation [47], angiogenesis, and regulation of gene expression [48]. Overexpression of anti-apoptotic proteins may drive carcinogenesis [49,50]. Anti-apoptotic Bcl-xL was involved in oral cancer progression as well as cisplatin resistance [51].

## 3. Discovery of Bcl-2

The oncogenic perspective on Bcl-2 was primarily proposed in 1988 by Reed et al. utilizing gene transfer technology [52]. *Bcl-2* gene rearrangements were explained to be linked with reduced prognosis in large-cell non-Hodgkin’s lymphomas (NHL) [53]. The study of chromosomal rearrangements, which occur in human cancers, has been the most potent and exciting approach for analyzing the molecular systems that trigger tumor cell growth. The examination of the t(14:18) translocation breakpoint and the finding of the *Bcl-2* gene had a unique impact on our thoughts on fundamental cell biology in malignant and normal cells [54,55,56]. Moreover, t(14:18) chromosomal translocations were observed via PCR in several normal individuals [57,58]. The Bcl-2 proto-oncogene was initially cloned from the t(14;18) translocation breakpoint in human follicular B cell lymphoma [3]. It is encoded by the *Bcl-2* gene in the case of the human genome, and it is specified as an oncogene [56]. It was identified due to its involvement in t(14;18) chromosomal translocations examined in non-Hodgkin’s lymphomas [56]. In translocations t(14;18), the *Bcl-2* gene is present on chromosome 18, which becomes combined with the immunoglobulin heavy-chain (IgH) locus present at chromosome 14. 

Effects in transcriptional activation of *Bcl-2* gene and the over-production of its protein occur in B-cells. Hence, Bcl-2 was expected to present another growth-inducing oncogene while it appeared to start the translocation breakpoint sequence. Bcl-2 was established to increase cell growth and obstruct cell death [3,59]. A study of experimental retrovirus-induced Bcl-2 overexpression in the mouse bone marrow cells showed that Bcl-2 over-expression alone could not be sufficient for tumorigenesis [3,59]. Increased Bcl-2 protein expression can certify the perseverance of a cell clone through the more active *Bcl-2* gene, awaiting promotion of tumorigenic alterations or mutations appearing in the result. The introduction of the cDNA of Bcl-2 in IL-3-dependent lymphoid and myeloid cells endorsed cell growth in the absence of the cytokine by the cells being sustained in a G0 position [3,59]. Experiments among transgenic mice, which overproduced Bcl-2 in B-cells, have powerfully confirmed the theory that inhibition of apoptosis characterizes one of the crucial steps in the process of tumorigenesis credibly [60,61]. The results achieved with mouse models are similar to the detected impact of the expression of Bcl-2 in cancers. Chromosomal Bcl-2 translocations affecting overexpression of protein are observed in follicular center B-cell lymphoma and CLL. These cancers are benign and consist of primarily quiet, non-cycling cancer cells [62,63]. 

## 4. Bcl-2 Structure and Function

Bcl-2 is a 26 kDa protein that is present on the mitochondrial outer membrane. However, it is also found on the ER membrane and the nuclear envelope [22], where it plays a pivotal role in promoting cellular growth, survival, and apoptosis. The *Bcl-2* gene encodes Bcl-2 in the human genome, specified as an oncogene [56]. It is ~250 kb in length and comprises three exons and two promoters [3]. This gene activates by the chromosomal translocation mechanism in various human cancers [64,65]. Bcl-2 was the first protein to be identified among Bcl-2 family proteins. Bcl-2 has two isoforms, Bcl-2α as well as Bcl-2β; Bcl-2α is anti-apoptotic [66].

**Figure 2:**
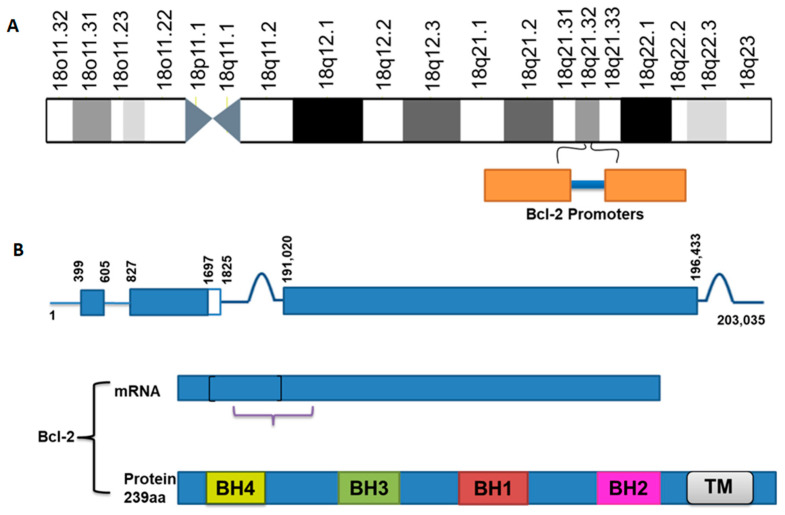
Structural composition of Bcl-2.(**A**) Figure indicating the position of the *Bcl-2* gene and promoters in the human chromosome. (**B**) The figure shows the *Bcl-2* gene composed of three exons, exon 1 and exon 2 encoding the four BH (BH1-4) domains and exon 3 encoding the transmembrane (TM) domain.

Bcl-2 is triggered by many transcription factors, including p53 [67], AP1, NF-κB, NFAT, and CRE [68]. Two promoter regions were documented in the 5′-untranslated region (5′-UTR) of the *Bcl-2* gene (Figure 2A). Moreover, the first promoter is situated 1386 to 1423 bp upstream of the translation starting site. It is the main transcriptional promoter [69,70], while the second promoter is situated 1.3 kb downstream from the first promoter near the first exon [69,70]. Regulations of Bcl-2 through NFκB, as well as AP-1, are vital in oral cancer and chemoresistance [71]. The major breakpoint region (MBR) in the 3′-UTR of the *Bcl-2* gene is about 200 kb downstream of the promoter that is a transcriptional regulatory element, which induces *Bcl-2* gene activity [72]. The regulatory role of the MBR is very much related to special AT-rich sequence binding protein 1 (SATB1) [48]. The 279-bp MBR in the 3′-UTR of the *Bcl-2* gene is a binding site of the SATB1, which is well identified as involving gene regulation [73]. The upstream region (21603 to 21579) of the promoter of the *Bcl-2* gene is composed of two GC/GA rich sites on 21601 (5′-GGGCTGG-3′) as well as 21588 (3′-GGAGGG-5′), which bid Sp1 protein [74]. The *Bcl-2* gene contains three exons; exon 1 and exon 2 encode the four BH domains, while exon 3 encodes the TM domain, which attaches the protein to intracellular membranes (Figure 2B) [69,75].

**Figure 3:**
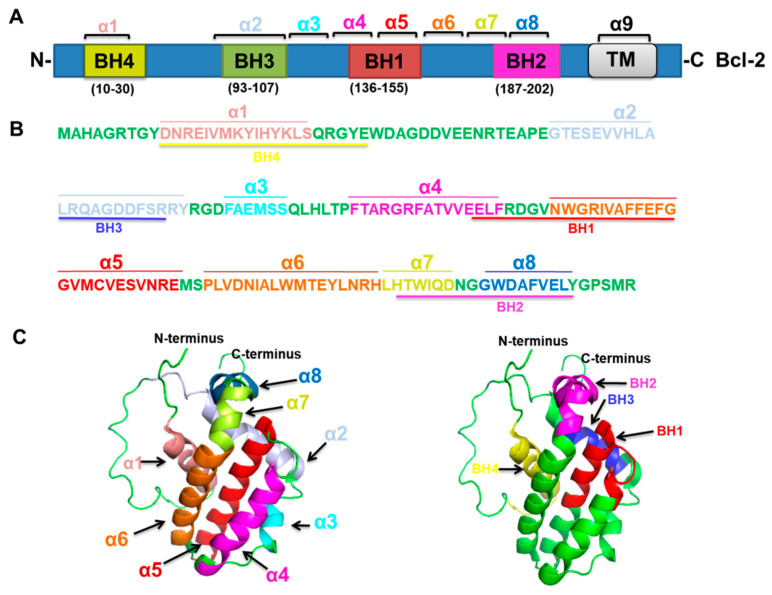
Bcl-2 amino-acid sequences and three-dimensional structure. (**A**) Bcl-2 is a 26 kDa, 239 amino-acid protein that is composed of four domains, the BH4 domain (residues 10–30), BH3 domain (residues 93–107), BH1 domain (residues 136–155), and BH2 domain (residues 187–202). (**B**) The amino acid sequences of the BH1-4 domains of Bcl-2. The colored letters denote various α-helices α1–α8 (Hα1–Hα8) and BH1-4 domains of Bcl-2. (**C**) The tertiary structure of Bcl-2; highlighted regions denote α-helices (α1–α8) and BH1-4 domains. The left panel indicates the Bcl-2 tertiary structure with α1–α8 with various colors, showing the same color coding as (**B**) (PDB ID: 1G5M). The right panel indicates the structure of Bcl-2 with the BH1-4 domains.

Bcl-2 protein contributes to the BH domain’s common genetic region and contains four conserved BH domains [32,37]. Bcl-2 is a 26 kDa, 239 amino-acid protein that composes four domains, BH1, BH2, BH3, and BH4 [38,76]. The BH4 domain residues (10–30), BH3 domain residues (93–107), BH1 domain residues (136–155), and BH2 domain residues (187–202) [76] (https://www.uniprot.org/uniprot/P10415#structure; accessed on 15 August 2021) (Figure 3A) and the amino acid sequences of the BH1–4 domains of Bcl-2 [76] (Figure 3B) are provided. The structure of a Bcl-2 chimeric protein comprising a truncated loop obtained from Bcl-xL among the Hα1 as well as Hα2 was initially observed through NMR spectroscopy [77]. Bcl-2 shows a tertiary structure (Figure 3C) containing two hydrophobic α-helices (Hα5 as well as Hα6) enclosed via amphipathic α-helices [77], since from N-terminus to C-terminus the Hα1 to Hα8 are attached one by one with/without a loop. However, the BH4 domain is well conserved at the N-terminal domain of Bcl-2, containing a length of 20 amino acids (10–30, residues) arranged in an α-helical construction. Therefore, BH1, BH2, and BH3 domains make a hydrophobic groove where BH3-only proteins may bind since mutations in this area were observed to stop the anti-apoptotic role of Bcl-2; the deletion of the Bcl-2-BH4 domain causes loss of the ability to bind BH3-only proteins [78,79]. Bcl-2 attaches to Bax by its BH1 and BH2 domains; this interface is vital to its function in regulating apoptosis, as shown in cells responding to cellular stress [5]. Bcl-2 is up-regulated and able to bind with Bax, which reduces apoptosis [66]. Bcl-2 BH1-2 domains are required for the inhibition of apoptosis [5]. The Bcl-2 protein binds and suppresses NALP1, decreasing caspase activation and interleukin-1beta (IL-1beta) production. The loop between BH4 and BH3 is needed for interaction with NLRP1 [80]. Bcl-2 was established to target Raf-1/ MAPK/ ERK for mitochondria in cells. Targeting the dynamic Raf-1 for the mitochondria can induce resistance for staurosporine-mediated cell death by interface with Bcl-2 [81]. Bcl-2 BH4 domain may attach directly to c-Myc, which promotes genetic instability, DNA damage, and tumorigenesis; hence, Bcl-2 is crucial to increased c-Myc transcriptional action and repression of DNA repair [82].

## 5. Bcl-2 Mediated Cancer Development

Bcl-2 plays a pivotal role in promoting cellular growth and survival that blocks pro-apoptotic Bcl-2 family proteins, which inhibit apoptosis [83]. Over-expression of the *Bcl-2* gene is involved in cancer development [1,2]. Bcl-2 represses apoptosis by blocking the release of cyt-*c* from the mitochondria, inhibiting the activation of caspases responsible for apoptosis [5]. The increased expression of Bcl-2 is linked with advanced-stage neoplasms and poor differentiation [7]. Elevated expression of Bcl-2 is involved in various cancer progression [6]. The increased regulation of Bcl-2 protects drug-induced cells from apoptosis [84]. Upregulation may happen via several mechanisms, including gene amplification, chromosomal translocation, gene/ protein expression and more. These mechanisms are involved in the regulation of Bcl-2 mediated cancer progression [85]. Paclitaxel may reverse resistance for cisplatin, initiating phosphorylation of Bcl-2, which facilitates cell death. Bcl-2 plays a vital role in angiogenesis as well as cancer growth [8]. Bcl-2 blocks TRAIL-induced cell death in glioblastoma, neuroblastoma, and breast carcinoma cells [86]. 

In the apoptotic pathway, inactive p53 fails to promote cell death through the interference disruption of MMP, disrupting the Bcl-2/Bax ratio [87]. p53 is a controller of the expression of the *Bcl-2* gene. It increases Bcl-2 protein expression [18,19,20]. The loss of normal p53 function results in diminished Bax expression [20]. Bcl-2 is the essential molecule whose phosphorylation and high expression involve controlling cell survival, proliferation, DNA repair, cell cycle, and tumorigenesis. Bcl-2 has been associated with several human cancers [13,14]. Upregulation of Bcl-2 protein certifies which over-proliferating cells grow when acquiring mutations or inducing tumorigenesis [88]. Endogenous Bcl-2 expressed in many cells may be phosphorylated on various sites in the flexible loop domain (FLD) such as Thr69, Ser70, and Ser87, which is linked with regulation of cell death [89]. Bcl-2 phosphorylation on Ser70 through growth factor-activated protein kinases, including PKC, may positively trigger the anti-apoptotic role of Bcl-2 [15,16]. The capability of Bcl-2 to balance the action of healthy characterized oncoproteins such as Myc underlies an extremely pivotal theory in cancer biology [90]. First, oncogenic mutations in genes resulting in out of control cell survival certainly activate safety machinery, which causes the removal of the mutated cells [91]. 

## 6. Targeting of Bcl-2 as a Novel Anticancer Treatment 

As we previously described in the earlier sections, dysregulated Bcl-2 family protein expression initiates the oncogenic transformation of healthy cells. Multiple Bcl-2 family proteins, Bcl-2 and Bax, are frequently established as over-or underexpressed in cancer tissue. The neoplastic cells can remain resistant to several apoptotic cues, which generates vital prerequisites to grow those eventual manifestations of tumors recognized as metastasis. Presently, anti-tumor protocols exploited in the clinic like radio- and chemotherapy aim to remove quickly proliferating cancer cells through “encouraging” completion of their mitochondrial apoptotic pathway. Overexpression of Bcl-2 via the reduction of Bax expression has been associated with the acquired resistance of cancers to radiation and chemotherapy. Hence, functional obstruction of anti-apoptotic Bcl-2 proteins or overexpression of pro-apoptotic Bcl-2 proteins might restore apoptosis in cancer cells and sensitize these cancers for chemotherapies and radiotherapies.

Due to the prominent function of Bcl-2 in inhibiting apoptosis, it has been identified as a potential target for the development of novel anti-tumor therapeutics. The improvement of novel anticancer therapies targeting Bcl-2 protein and Bcl-2 family proteins is an aim followed via several research groups, and their job entails three key strategies: (i) Decreasing regulation and expression of the anti-apoptotic Bcl-2 family proteins by targeting their mRNAs; (ii) Interference with the role of anti-apoptotic Bcl-2 family proteins on the protein stage by utilizing Bcl-2-attaching compounds; (iii) Induction of cell death via the introduction of pro-apoptotic proteins. The first aim, particularly targeting Bcl-2 family proteins, is based on the reality that antisense oligonucleotides can hybridize for target mRNAs, leading to their degradation after reducing *de novo* synthesis of Bcl-2 protein. Based on the high homology of some Bcl-2 and Bcl-xL mRNA regions, it was tried to plan antisense oligonucleotides by double specificity, targeting the two main anti-apoptotic proteins engaged in tumorigenesis. Pre-clinical study of antisense oligonucleotides indicated concurrent downregulation of Bcl-2 and Bcl-xL and cell death induction in several cancers in vitro and in vivo [92,93,94]. 

To hinder the role of Bcl-2 in the protein stage, Wang et al. [95] proposed other approaches where small molecules attach and inactivate the overexpressed Bcl-2 protein. As a scientific study showed, a cell porous Bcl-2 attaching peptide was planned through chemically binding a fatty acid for a peptide obtained from Bax, which blocked survival of Bcl-2 transfected HL-60 cell lines during in vitro and in vivo studies. Small-molecule inhibitors were documented which attach to Bcl-2 and Bcl-xL, interrupting the interface between Bcl-2/Bcl-xL and the BH3 domain, thereby inducing apoptosis [96,97]. Hence, the cell’s decision to go through cell death depends, among other things, on the percentage of pro-apoptotic and anti-apoptotic Bcl-2 proteins; through increased regulation of pro-apoptotic proteins, this has been attempted for apoptosis induction in cancer cells. Thus, Bax expression in LNCaP affected an 85% decrease in cell viability by induction of cell death, showing the potency of a similar strategy [98]. The therapeutic function of peptides tolerates many problems; hence small agents mimicking particular BH3 domains could make excellent tools for clinical trials. More current approaches for targeting Bcl-2 include exploiting inhibitors that mimic BH3 composing proteins and attach particularly in the hydrophobic groove at the anti-apoptotic proteins since these small molecules are also known as BH3-mimetics.

## 7. Bcl-2 Inhibitors

**Figure 4:**
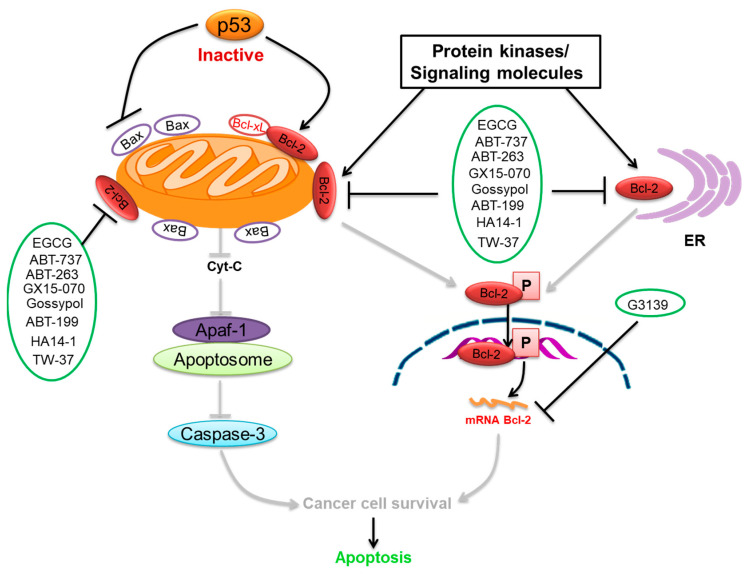
Bcl-2-mediated apoptosis cascade and the mechanism of action of inhibitors. Over-expression of Bcl-2 might be blocked and inhibited through Bcl-2 inhibitors. Inhibitors block cancer progression by targeting signaling molecules or protein kinases that abrogate Bcl-2 expression and decrease the expression of Bcl-2 by inhibiting cell growth and proliferation and initiating apoptosis.

We summarize here the critical Bcl-2 inhibitors from published patents and literature (Table 1). Bcl-2 inhibitors may inhibit cancer cell growth and survival (Figure 4). These inhibitors were reported in the patents as well as literature databases.

### 7.1. Oblimersen

Oblimersen (G3139) is a phosphorothioate Bcl-2 antisense oligodeoxynucleotide, which targets the mRNA of Bcl-2. The apoptosis mechanism of Bcl-2 antisense may be grouped into two types, apoptotic and nonapoptotic, based on their apoptotic ability. G3139 antisense increases Bax and PARP, which releases cyt-*c* for activating caspases and releases Smac/DIABLO for antagonizing inhibitors of cell death proteins from mitochondria for stimulating DNA fragmentation [119]. Inhibitors activate caspase-3 as well as caspase-9 that initiates cell death. Bcl-2/Bcl-xL interacts with the BH3 domain of Beclin-1, where pharmacological disturbance of the interaction between Beclin-1 and Bcl-2 may induce autophagy [120]. Bcl-2 decreased regulation through Bcl-2 antisense and was identified to stimulate autophagic cell death within HL-60 cells, possibly by releasing Beclin-1 [121]. Oblimersen was clinically studied in combination with other anti-tumor chemotherapeutic drugs in multiple cancers [99,100]. Oblimersen has studied in current phase II as well as phase III clinical trials. Oblimersen was designed to target Bcl-2 as well as Bcl-xL. Some compounds have indicated attractive clinical study in phase I and II trials, particularly when combined with other cytotoxic drugs [122,123]. Anti-Mcl-1 AON reduced cell viability in vitro in combination with bortezomib and chemotherapy [124].

### 7.2. Gossypol

Gossypol (AT-101), a natural compound, was extracted in 1915 from cottonseed [125]. It has been widely documented since the 1980s, like contraceptives and anti-tumor drugs [126,127]. Gossypol has anti-tumor activity because of extensive results on cells, which trigger Bcl-2 protein and caspases [128], DNA damaging capability, and trigger p53 [129]. It was initially exploited to examine glial cancers; however, its mechanism of action was unidentified at that time [130]. Gossypol occurs inside the racemic shape, and Levo isoform is presently in clinical trials [131]. Levo isoform was indicated to be highly effective in its survival inhibitory results. Multi-dimensional NMR techniques have observed Levo isoform binding with the hydrophobic groove of Bcl-xL and Bcl-2 [132], which generates ROS and cytochrome c release [133]. Gossypol is presently in pre-clinical study. AT-101 shows submicromolar attaching affinity to Bcl-2 as well as Mcl-1. However, gastrointestinal toxicity has been dose preventive in Phase I and II clinical studies for prostate cancer. Apogossypol is in pre-clinical improvement. Apogossypol looks for superior targets on Bcl-2 and Mcl-1 that can reduce the systemic toxicities detected with gossypol.

### 7.3. Obatoclax

Obatoclax (GX15-070) is an indole bipyrrole complex, which antagonizes Bcl-2, Mcl-1, Bcl-xL and Bcl-W [134]. Obatoclax was proposed after researchers discovered Bcl-2 proteins, which have promise for conformational alterations; they utilized high throughput screening of natural amalgam libraries, disturbing protein-protein interactions [135]. It can block the direct interface among Mcl-1 and Bak that was observed to overcome the resistance for ABT-737 and bortezomib [136]. Obatoclax may regulate cell death in NSCLC and might increase cisplatin-based therapy-mediated death [137]. In a pre-clinical trial, it was indicated for stimulating promising cytotoxic reactions against cancer cells and increasing the antimyeloma action induced through bortezomib [138]. Obatoclax was observed for stimulating Bax-mediated cell death in cholangiocarcinoma [132]. Hence, this compound has indicated activity against several tumor cells, including melanoma, esophageal cancer cells, and PC cells [114,115]. Obatoclax increased TRAIL-induced apoptosis, as observed via the Annexin V group accompanied by activation of caspase-8, -9, and -3, and cleavage of Bid. Hence, it potentiated TRAIL-induced Bak/Bax activation, releasing cyt c, Smac, and AIF. These mechanisms were primarily the apoptotic result of obatoclax, including, dislocation of Bak from its confiscation via Bcl-xL/Mcl-1 that liberates Bim from Bcl-2 [139]. Pre-clinical trials indicated that obatoclax has single-drug activity and increased in vitro cytotoxicity of bortezomib against several myelomas [136].

### 7.4. EGCG

Epigallocatechin-3-gallate (EGCG) is the most abundant and closely examined polyphenol in green tea [140,141]. The chemotherapeutic effects of EGCG have been documented against various cancers [142,143,144]. EGCG alters and inhibits the Bcl-2 family protein ratio and activates caspases in cancer cells [145,146,147]. It has anticancer effects that increase Bax and Bak and decrease Bcl-xL and Bcl-2, initiating activation of caspases-9 and inducing cell death in SCC cancer cells [148,149]. EGCG leads to down-regulation of Bcl-2 and Bcl-xL [150]. The interaction of EGCG with p53 disrupts p53 with its regulatory E3 ligase MDM2. It reduces the ubiquitination of p53 through MDM2, since EGCG interrupts the binding of p53 for its regulator MDM2, stabilizing p53 through blocking p53 ubiquitination as well as degradation [151]. EGCG was documented from a library of some 2295 phytochemicals as an inhibitor of p53 with MDM2 interaction [152]. 

### 7.5. HA14-1

HA14-1 is an inhibitor of anti-apoptotic proteins, which was documented via structure-based screening [115]. HA14-1 stimulated cell death in several tumor cells [153]. It increased the cytotoxicity of doxorubicin, TRAIL ligand, bortezomib, and flavopiridol, resulting in a growth stoppage in the glioblastoma xenograft model [153]. The attaching affinity of HA14-1 for Bcl-2 is comparatively elevated compared to inhibitors of anti-apoptotic Bcl-2 proteins [115]. HA14-1 decomposes quickly within the solution, while a chemical alteration has developed its constancy [154]. The HA14-1 ligand of the Bcl-2 protein surface pocket was proposed [155]. This molecule has exposed the activity of several tumor cell lines [156,157]. HA14-1 is unbalanced on physiological conditions. However, it crumbles quickly and produces ROS, which might be a mediator of apoptosis, and this compound must be utilized carefully like a qualified opponent against anti-apoptotic proteins [158]. 

### 7.6. ABT-737 

ABT-737 is a potent inhibitor of Bcl-2 and Bcl-xL [159], and its application in cancer therapeutics has been observed [104]. ABT-737 has its effect on activating caspase-3, leading to cell death. ABT-737 up-regulates pro-apoptotic Noxa expression via synergistic combination with chemotherapy [104]. ABT-737 binds with a very high affinity to Bcl-xL/Bcl-2 because of their similar structure [105]. Hence, Bcl-2 is inhibited due to the binding of ABT-737 in its hydrophobic groove, which relocates any bound pro-apoptotic BH3-containing proteins [105]. ABT-737 stimulated the caspase-3 activation as well as cleavage of PARP, which induced cell death. ABT-737 decreased the activation of Akt expression, which is involved in this signaling pathway in the inhibition of gastric cancer cell growth. p53 was observed to be linked to the effect of ABT-737 as well as naringenin in gastric cells [160]. Data showed ABT-737 and epothilone B caused a blockade of the signaling pathways that could synergistically stimulate apoptosis and anti-proliferation in human cancer cells [161]. 

### 7.7. ABT-263

ABT-263 (Navitoclax) shares analogous biological characteristics with ABT-737. It acts as a single drug in SCLC xenografts; it increased the activity of other therapy drugs in pre-clinical trials of B-cell lymphoma and multiple myeloma [107]. It is in a clinical study for chronic myelogenous leukemia as well as SCLC in adults. ABT-263 is orally bio-available and attaches to Bcl-2, Bcl-xL, as well as BCL-w [107]. The biological action of ABT-737 and ABT-263 is shown to be similar; ABT-263 was observed to be more highly sequestered via human serum albumin than ABT-737 [162]. ABT-263 has been in clinical trials for lymphoid malignancies and solid tumors. However, while the effects in hematological cancers were promoted and single-drug activity was identified [106], the efficiency of ABT-263 like a single drug in solid tumors was a little unsatisfactory. In the Phase I dose-escalation trial, 47 patients were registered; 27 had SCLC [163]. The pre-clinical data of SCLC patients reacted better than the other cancers. In the phase II trial, patients with SCLC were established on a lead-in dose of 150 mg daily for one week pursued via 325 mg of ABT- 263 daily [164]. Navitoclax-induced cytotoxicity engages the destruction of interactions between Bcl-2/Bcl-L, BIM, and Bax translocation and consequently liberates cyt*c*, activating apoptosis. Hence, this mechanism was detected to be caspase-dependent [107]. It has been assessed frequently in combination with other agents, particularly in solid tumors [105]. In phases I and II, trial studies were performed in patients with refractory CD20+ lymphoid tumors [165]. 

The main toxicity of ABT-263 was an on-target result of Bcl-xL expressed in platelets [166,167,168]. The finding that thrombocytopenia was a key mechanism-based result of ABT-263 led to studies that exhibited the significance of Bcl-xL as a molecular clock in platelets [166]. Pharmacological inactivation of Bcl-xL decreases platelet half-life and causes thrombocytopenia in a dose-dependent way [166]. The antagonistic equilibrium between Bcl-xL and Bak comprises a molecular clock, which decides platelet life span; this shows an essential paradigm of cellular homeostasis that has profound suggestions for the diagnosis and treatment of disorders that affect platelet number as function [166]. Treatment with ABT-263 stimulates a selective loss of older platelets, which justifies the transient thrombocytopenia detected with ABT-263 treatment [167]. The dose-limiting severe thrombocytopenia from ABT-263 quenched the interest in further clinical improvement of this compound. Pre-clinical studies of ABT-737 also revealed decreased platelet survival [169]. Hence, Bcl-xL inhibition might speed up the molecular clock and cause reduced platelet survival, the mechanism associated with ABT-263/ABT-737-induced thrombocytopenia.

### 7.8. ABT-199

ABT-199 (Venetoclax) is a promising and selective inhibitor of Bcl-2 protein, which has exhibited clinical efficacy in multiple hematological cancers [170]. ABT-199 is an inhibitor that exclusively maintains binding for Bcl-2 [171]. ABT-199 efficiently stimulates cell death in Bcl-2-dependent cancers without inducing thrombocytopenia. A single dose of ABT-199 stimulated cancer lysis syndrome in leukemia, showing potent anticancer action in vivo in humans. It is presently one of the most stimulating drugs for hematological cancers in clinical progress. Due to the important function of Bcl-2 in B-cells, clinical studies with ABT-199 presently concentrate exclusively on hematological cancers. A selective inhibitor of Bcl-2 can induce apoptosis. SCLC exhibits an elevated expression of Bcl-2 and can be liable to single-drug treatment by ABT-199. Through overexpression of Bcl-xL, ABT-199 showed pre-clinical trial activity in breast cancer cells [109] by investigation of the promise of ABT-199 as a Bcl-2 inhibitor.

Venetoclax represents the first-in-class selective, Bcl-2 inhibitor sparing platelets [169]. It exhibited a sub-nanomolar affinity for Bcl-2 with anticancer activity against NHL [63]. In studies, the Venetoclax resistant cell was recognized, and enhanced levels of Mcl-1 and elevated phosphorylation of Bcl-2 at T56 and AKT at S473 were shown. A single dose of venetoclax in patients with refractory CLL resulted in cancer lysis within 24 h. Hence, pharmacological inhibition of Bcl-2 indicates potency for the treatment of Bcl-2-dependent cancers [169]. One study emphasizes Bcl-2 as a molecular target in particular subtypes of human T-ALL, which might be exploited via ABT-199 [172]. The novel combination of venetoclax with decitabine was efficient and well-tolerated in elderly patients with AML [173]. Venetoclax was examined in combinations with tyrosine kinase inhibitors (TKIs), including imatinib, dasatinib, and nilotinib in cells from six patient samples with blast-crisis CML [70]. All six samples were resistant to TKIs, three of them with T315I mutation. In a CML mouse model, the study further revealed that venetoclax alone or in combination was better than nilotinib in eliminating CML stem cells in vivo. To analyze the dual inhibition of Mcl-1 and Bcl-2, HHT and venetoclax were combined and investigated in seven diffuse large B-cell lymphoma cell lines [174]. Presently, venetoclax is being investigated in >230 clinical studies in a broad range of hematological cancers. Venetoclax has exhibited clinically consequential single-drug activity in selected lymphomas [175].

### 7.9. TW-37 

TW-37 is a potential anti-tumor drug that has been identified in the prostate as well as pancreatic cancer [176]. TW-37 is a benzene-sulfonyl derivative, which originated from gossypol. One laboratory has broadly analyzed TW-37 for its activity in lymphoma, leukemia, and PCs [175,176], exhibiting anti-angiogenic activity [177]. TW-37 has a high affinity to Bcl-2. TW-37 stimulates cell death in PCs by a novel NOTCH-1 signaling pathway [178]. It is a promising inhibitor of the development of PCs because it reduces the Bcl-2 cellular pathway mechanism. TW-37 was developed as a potential therapeutic drug for the treatment of PC [139]. The anticancer activity of ApoG2, as well as TW-37, was observed to be induced by a novel pathway linking induction of PAR-4. SMI-induced cell death engaged PAR-4 in PC [179]. The detected anticancer activity of TW-37 is induced by a novel signaling pathway linking the inactivation of NOTCH-1 as well as Jagged-1 [178]. TW-37, a Bcl-2 inhibitor, exhibited synergistic results against SCC and cancer-connected endothelial cells in vivo and in vitro combined for cisplatin. Hence, it induced cell death and reduced angiogenesis, improving time for cancer failure [110].

## 8. Limitations of Bcl-2 Inhibitors

The limitations of the first Bcl-2 family protein-targeted drugs, concerning on-target and off-target toxicities, have been overcome with the improvement and development of venetoclax [180]. ABT-737 development has been limited, and navitoclax, its orally bioavailable analog, has been developed. ABT-263 exhibited efficacy in vivo in xenograft models of leukemia as well as lymphoma [181]. Beginning from the natural compound/agent like meiogynin A, molecules particularly targeting Bcl-2/Mcl-1 [174], have been produced, synthesized, and tested in vitro, however, with limited application. Pre-clinical data supports the Bcl-2 G-quadruplex (G4)-selective move toward treating cancer and circumventing the limitations of Bcl-2 protein-based therapeutics [182]. Early generation Bcl-2 inhibitors have exhibited potency in the clinic; resistance is anticipated, as detected in several in vitro models. Hence, this resistance may occur from the up-regulation and dependence on extra anti-apoptotic proteins, which are not blocked via ABT-263/ABT-199 [183,184]. Incubation of CLL cells on stroma stimulates dramatic resistance for ABT-199 and ABT-737 that may be circumvented through combination with gossypol/ AT-101 [185,186]. Resistance for ABT-199 might arise from up-regulation of other Bcl-2 proteins; resistance may be mimicked via culturing CLL cells on CD154(+) stromal cells [185]. Venetoclax monotherapy for an extended duration may cause drug resistance or loss of dependence on the targeted protein [187]. Upregulation of MCL-1 has appeared as a general determinant of venetoclax resistance [188]. Resistance in lymphoid cells for ABT-199 has been documented [189]. Some diseases are liable to be as dependent on one Bcl-2 protein as CLL; the majority of tumors will likely require combinations of these drugs to have a significant therapeutic impact; however, these combinations may show unacceptable toxicity [190,191,192,193,194,195,196,197,198,199,200,201].

## 9. Conclusions and Future Directions

Bcl-2 plays a central role in the regulation of apoptosis that is a major contributor to cancer development. Bcl-2 has been identified as being over-expressed in several cancers. Regulation of Bcl-2 by multiple signaling pathways, including p53, protein kinases, and signaling molecules, induces cancer development. Consistent with this examination, drugs and agents mimic major modulators of cell death that have appeared to produce positive results over the last many years. The success of any therapeutic agent depends primarily on the capability of therapeutic tools for activating cell death through targeting the anti-apoptotic proteins and initiating the expression of pro-apoptotic proteins. Bcl-2 has been recognized as a potential target for the development of novel anti-tumor drugs. However, Bcl-2 inhibitors have been shown to down-regulate Bcl-2 and up-regulate Bax, initiating apoptosis.

Several agents and drugs have been designed for targeting Bcl-2 at the mRNA and protein level. Pharmacological and cellular features of drugs targeting Bcl-2 could be found while exploring their prospective applications, such as chemotherapy. However, the binding affinity for blocking anti-apoptotic proteins should optimally be in clinically possible concentrations for each drug. Hence, the big challenge is investigating how best to utilize these inhibitors and which tumor types. The inhibitors of Bcl-2 are efficient as single agents, and they might be extremely beneficial when combined with additional targeted agents which induce cell death in cancer cells. Increasing the therapeutic results of Bcl-2 inhibitors of anti-apoptotic Bcl-2 protein should be done using a combination of agent which target various Bcl-2 family proteins. These treatment strategies can restore the Bcl-2 mediated apoptosis towards normality, potentially eliminating cancer cells.

Some strategies for blocking pro-survival and overexpressed Bcl-2 have been advised through the medical literature, looking at these many clinical trials. Presently several Bcl-2 inhibitors and antisense are being studied in clinical trials. Pre-clinical trials appear promising, particularly in combination with other therapeutic drugs. Continuing and designed phase II trials for describing the action of single drug and agent combinations will conclude future clinical improvement and development of Bcl-2 inhibitors.

Bcl-2 PROTAC (proteolysis targeting chimera) employs an E3 ligase for a target protein to induce its ubiquitination and degradation that potently and selectively induces the degradation of Bcl-2 and Mcl-1. PROTACs indicated reversible depletion in living cells that provide a novel promising toolkit for gain-of-function examinations to probe the dynamic functions of Bcl-2 as well as Mcl-1 in apoptosis. Hence, particular degradation of a target protein using the PROTAC for overcoming on-target drug toxicity. However, two PROTAC compounds have been reported to degrade Bcl-2/Mcl-1 selectively.

## Figures and Tables

**Table 1:** Bcl-2 inhibitors are used for the therapeutic targeting of several cancers.

Agents	Chemical Structure	IC_50_ for Bcl-2 (μM)	IC_50_ for Bcl-xL (μM)	Used for Treatment	Clinical Status	References
G3139	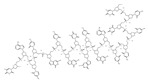	NA	NA	Solid Tumor (ST), SCLC, Melanoma Leukemia, etc.	Phase 1	[99,100]
EGCG	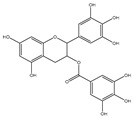	0.45	0.59	HNSCC, OSCC, other cancers	Phase 1/2	[101,102,103]
ABT-737	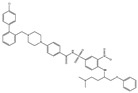	0.12	0.064	HNSCC, ST, PC, Leukemia, etc.	Phase 1/2	[101,104,105]
ABT-263	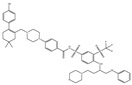	NA	NA	ST, Haemato logical malig- nancies, SCLC	Phase 1/2	[106,107]
ABT-199	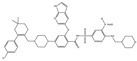	0.1	NA	ST, Breast cancer	Approved for use in CLL	[108,109]
TW-37	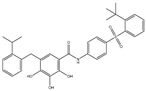	NA	NA	HNSCC, Prostrate Cancer, PC,	Phase 1/2	[110,111]
Gossypol	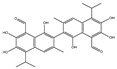	0.28–10	0.4–3.03	HNSCC, ST, PC	Phase 1/2	[101,112]
GX15-070 (Obatoclax)	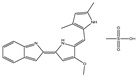	NA	NA	HNSCC, PC, ST, NSCLC	Phase 1	[113,114]
HA14-1	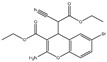	~9	NA	HNSCC, leukemia, lymphoma, colon cancer, etc.	Pre-clinical	[113,115,116]
Chelerythrine	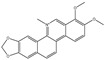	~10	~10	HNSCC, ST, etc.		[101,117]
S55746	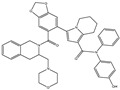	NA	NA	Hematological tumor	Phase 1	[118]

## Data Availability

Not applicable.

## Abbreviations

Bcl-2	B cell lymphoma 2
BH	Bcl-2 homology domain
MMP	Mitochondrial membrane permeabilization
MBR	Major breakpoint region
CLL	Chronic lymphocytic leukemia
NSCLC	Non-small cell lung cancer
SCLC	Small-cell lung cancer
NHL	Non-Hodgkin lymphomas
SATB1	Special AT-rich sequence binding protein 1
IgH	Immunoglobulin heavy chain
TNF	Tumor necrosis factor
UTR	Untranslated region
PKC	Protein kinase C
EGCG	Epigallocatechin-3-gallate
